# Rapid neurophysiological screening for sensory ganglionopathy: A novel approach

**DOI:** 10.1002/brb3.880

**Published:** 2017-11-24

**Authors:** Panagiotis Zis, Marios Hadjivassiliou, Ptolemaios Georgios Sarrigiannis, Alexander St John Edward Barker, Dasappaiah Ganesh Rao

**Affiliations:** ^1^ Academic Department of Neurosciences Sheffield Teaching Hospitals NHS Foundation Trust Sheffield South Yorkshire UK; ^2^ Department of Clinical Neurophysiology Sheffield Teaching Hospitals NHS Foundation Trust Sheffield South Yorkshire UK

**Keywords:** neuronopathy, screening, sensory ganglionopathy

## Abstract

**Background and Aim:**

Pure sensory neuropathies involving the dorsal root ganglia are commonly referred to as sensory ganglionopathies (SG). Causes of SG can be inherited (as seen in Friedreich's ataxia) or acquired (e.g. immune‐mediated or paraneoplastic). Diagnostic criteria for confirming SG have been published and consist of a combination of clinical and neurophysiological parameters. The aim of our study was to develop a neurophysiological method for rapid screening for diagnosis of SG.

**Methods:**

For each subject we obtained the sensory nerve action potentials (SNAPs) of five nerves (median, ulnar, radial, sural and superficial peroneal) bilaterally. In the presence of an entrapment neuropathy we obtained the SNAP of the medial antebrachial cutaneous nerves bilaterally. We estimated the number of pairs of nerves showing a SNAP asymmetry of >50% (difference of SNAPs/ lower SNAP).

**Results:**

Sixty‐eight subjects, 34 patients with SG and 34 age and sex‐matched controls, participated in the study. Among all subjects using a receiver operating characteristic (ROC) curve analysis, the area under the curve was 0.984 (95% CI, 0.960–1.000; SE, 0.012; *p *< .001). In order to detect SG, presence of SNAP asymmetry of >50% in 2 pairs of nerves, not explained by an entrapment neuropathy, shows a sensitivity of 97.1%, a specificity of 94.1%, a positive predictive value of 94.3% and a negative predictive value of 97.0

**Conclusion:**

The number of pairs of nerves showing a SNAP asymmetry of >50% may be used as a novel rapid screening tool of patients with SG.

## 1 INTRODUCTION

Sensory neuronopathy also known as sensory ganglionopathy (SG) is a type of pure sensory neuropathy affecting the cell bodies of the sensory neurones located in the dorsal root ganglia (Zis, Sarrigiannis, Rao, Hewamadduma, & Hadjivassiliou, 2016). Clinically SG is characterized by asymmetric patchy sensory symptoms and/or sensory ataxia.

Causes of SG can be inherited (as seen as part of Friedreich's ataxia) or acquired (such as immune‐mediated or paraneoplastic). Common causes of immune‐mediated SG include Sjögren's syndrome (Pereira et al., 2016) and non‐celiac gluten sensitivity or celiac disease (gluten SG) (Hadjivassiliou et al., 2010; Zis, Rao, Sarrigiannis, et al., 2017). In the majority of the paraneoplastic SG, anti‐Hu seropositive small cell lung carcinoma is the commonest cause, but other forms of cancer have also been linked to SG (Zis, Rao, Wagner, et al., 2017). However, there are cases of SG that remain idiopathic despite extensive diagnostic work‐up and prolonged follow up (Zis et al., 2016).

Early diagnosis of SG is particularly important, as the full diagnostic work up can potentially identify a reversible cause. For example in paraneoplastic SG, early diagnosis of SG can lead to the diagnosis of the underlying malignancy at a treatable stage or in gluten SG, embarking on a gluten‐free diet is known to be protective (Hadjivassiliou et al., 2010).

Diagnostic criteria for sensory ganglionopathy have been published (Camdessanché et al., 2009) and consist of a combination of five clinical and neurophysiological parameters. The three clinical parameters are 1) presence of ataxia in the lower or upper limbs at onset or full development, 2) presence of asymmetrical distribution of sensory loss at onset or full development and 3) sensory loss not restricted to the lower limbs at full development. The two neurophysiological criteria are 1) at least 1 sensory potential absent or 3 sensory nerve action potentials (SNAP) <30% of the lower limit of normal in the upper limbs, not explained by entrapment neuropathy and 2) less than two nerves with abnormal motor nerve conduction studies (NCS) in the lower limbs. These criteria can characterize a patient as having a possible SG but when they are also combined with presence of anti‐neuronal antibodies or high signal in the posterior columns of the spinal cord the patient is characterized as having a probable SG.

These criteria, however, do not include the presence of SNAP asymmetry between the two sides, which in our experience is very common in SG and neurophysiologically may represent a hallmark of SG.

The aim of our study was to set up a neurophysiological method for rapid screening and diagnosis of SG.

## 2 METHODS

### 2.1 Study design

Data collected for the purpose of audit, service evaluation and quality improvement were used for this study. All subjects were examined clinically and neurophysiologically at the Department of Neurology and the Department of Clinical Neurophysiology in the Royal Hallamshire Hospital in Sheffield, UK respectively. The study was approved by the local audit committee.

### 2.2 Electrophysiological studies

The following parameters measured using a Natus electromyography were used for this study:

1. Median SNAP (orthodromic). The cathode and anode ring electrodes were placed in the proximal and middle phalanxes of the third digit, respectively. The active recording electrode was 2 cm proximal to the wrist crease, over the median nerve between the flexor carpi radialis tendon and the palmaris longus tendon. The reference recording electrode was placed 3 cm proximal to the active electrode.
2. Ulnar SNAP (orthodromic). The cathode and anode ring electrodes were placed in the proximal and middle phalanxes of the fifth digit, respectively. The active recording electrode was 2 cm proximal to the wrist crease, over the ulnar nerve just lateral to the flexor carpi ulnaris tendon. The reference recording electrode was placed 3 cm proximal to the active electrode.
3. Superficial radial SNAP (antidromic). The active recording electrode was placed over the anatomical “snuff box” formed by the extensor pollicis brevis and abductor pollicis longus tendons laterally and the extensor pollicis longus tendon medially. The reference recording electrode was placed 3 cm distal to the active electrode. Stimulation was 10 cm from the active recording electrode, over the dorsolateral edge of the radius bone.
4. Sural SNAP (antidromic). The active recording electrode was placed between the lateral malleolus and the Achilles tendon. The reference recording electrode was placed 3 cm distal to the active electrode. Stimulation was 10 cm proximal to the active recording electrode, over the distal posterolateral leg.
5. Superficial peroneal (fibular) SNAP (antidromic). The active recording electrode was placed over the dorsum of the foot at the level of the malleoli slightly lateral to the midline. The reference recording electrode was placed 3 cm distal to the active electrode. Stimulation was 10 cm proximal to the active recording electrode, over the distal anterolateral leg.
6. Medial antebrachial cutaneous SNAP (antidromic). The active recording electrode was placed over the medial forearm, 10 cm distal to the cathode on a line between the stimulation site and the ulnar styloid at the wrist. The reference recording electrode was placed 3 cm distal to the active electrode. Stimulation was 3 cm proximal to the midway point between the biceps tendon and the medial epicondyle of the humerus.

On all occasions supramaximal stimulation of the nerves was performed. The SNAP amplitudes were measured as base to peak. The following settings were also applied; filter 2 Hz‐2KHz, stimulus duration 0.2 ms, sweep speed 10 ms (1 ms/division).

For each participant we recorded the SNAPs of 5 pairs of nerves (median, ulnar, superficial radial, sural and superficial peroneal). Motor conduction studies were used to determine the presence of entrapment neuropathies. The SNAPs of medial antebrachial cutaneous nerves bilaterally were recorded only in patients with neurophysiological evidence of carpal tunnel syndrome (Zis et al., 2015) or ulnar entrapment neuropathy (Campbell, 1997) as a substitute parameter for the median or ulnar NCS respectively.

### 2.3 Participants

All patients presented with clinical symptoms and signs consistent with SG (i.e. absence of motor involvement and asymmetric patchy sensory symptoms or sensory ataxia). All patients fulfilled the diagnostic criteria for SG (Camdessanché et al., 2009). Further characterization of the underlying etiology of the SG was based on extensive serological and immunological screening, genetic testing for patients with family history suggestive of a genetic neuropathy and paraneoplastic screening (antineuronal antibodies and PET scan) for patients with sub‐acute or rapidly progressing symptomatology. As complete absence of SNAP with normal motor NCS is diagnostic of a severe SG (commonly such picture is seen in cases of Friedreich's ataxia or paraneoplastic syndromes) we included in the analysis only patients with recordable SNAPs in at least 1 nerve out of the 10 recorded in each case.

Control subjects were patients with length‐dependent sensorimotor axonal peripheral neuropathy (PN). Further characterization of the underlying etiology of the PN was based on extensive serological and immunological screening. We have also included subjects with no clinical evidence of peripheral neuropathy in the control group. Control subjects were age and sex matched to the patients with SG.

In all cases the duration of the symptoms prior to the nerve conduction studies was <12 months.

### 2.4 Statistical analyses

A database was developed using the Statistical Package for Social Science (version 23.0 for Mac; SPSS). Frequencies and descriptive statistics were examined for each variable. Comparisons between groups were made using Student's t‐test for continuous data and chi‐square test for categorical data.

We calculated the absolute difference between the two sides (right and left) in each of the 5 pairs of SNAP (higher SNAP minus lower SNAP) and we then converted it to a percentage (difference between the SNAPs/ lower SNAP). In order to identify the optimum level suggestive of asymmetry we analyzed our data at two levels of asymmetry; at a cut‐off of equal to or >50% (i.e. higher SNAP is at least 50% greater than the lower SNAP) and at a cut‐off of equal to or >100% or greater (i.e. higher SNAP is at least double the lower SNAP). We then calculated the number of pairs our of 5 examined, that showed asymmetry.

Receiver operator characteristics (ROC) analysis was calculated to assess the utility of the total number of asymmetries to distinguish the diagnosis of SG. Area under the curve (AUC) and its 95% confidence intervals (CI) for the ROC curve were calculated. The AUC is a measure of the diagnostic power of the test, independent of cut‐off points. An AUC < 0.60 is considered “negative”, 0.61 – 0.80 as “doubtful”, 0.81 – 0.90 as “good” and >0.91 as “very good” (Altman, 1999). The Youden Index was calculated as the sum of sensitivity plus specificity minus 1 for all possible cutoff points to identify the most relevant cutoff values (Youden, 1950).

A value of *p *< .05 was considered to be statistically significant.

## 3 RESULTS

In total data from 68 subjects, 34 patients with SG and 34 sex and age‐matched controls (20 patients with PN and 14 subjects with no PN) were used for this study. The demographic and clinical characteristics of the two groups are summarized in Table 1.

**Table 1:** Charactersitics of patients with SG and controls

	SG (*n *= 34)		Controls(*n *= 34)	
Age, in years (SD)^a^	66.5 (10.3)		63.9 (14.6)	
Male gender (%)^a^	15 (44.1)		16 (47.1)	
Cause of PN (%)	Gluten sensitivity/ CD	13 (38.2)	Gluten sensitivity/CD	5 (14.7)
	Sjogren's	8 (23.5)	DM	2 (5.9)
	Paraneoplastic	5 (14.7)	Idiopathic	10 (29.4)
	Idiopathic	6 (17.6)	Alcohol related	1 (2.9)
	RA	1 (2.9)	B12 deficiency	1 (2.9)
	FA	1 (2.9)	Uremic	1 (2.9)
			No PN	14 (41.2)

SG, sensory ganglionopathy; PN, peripheral neuropathy; CD, celiac disease; RA, rheumatoid arthritis; FA, Friedreich's ataxia.

a No statistically significant difference.

### 3.1 Asymmetry of 50% or more

**Figure 1:**
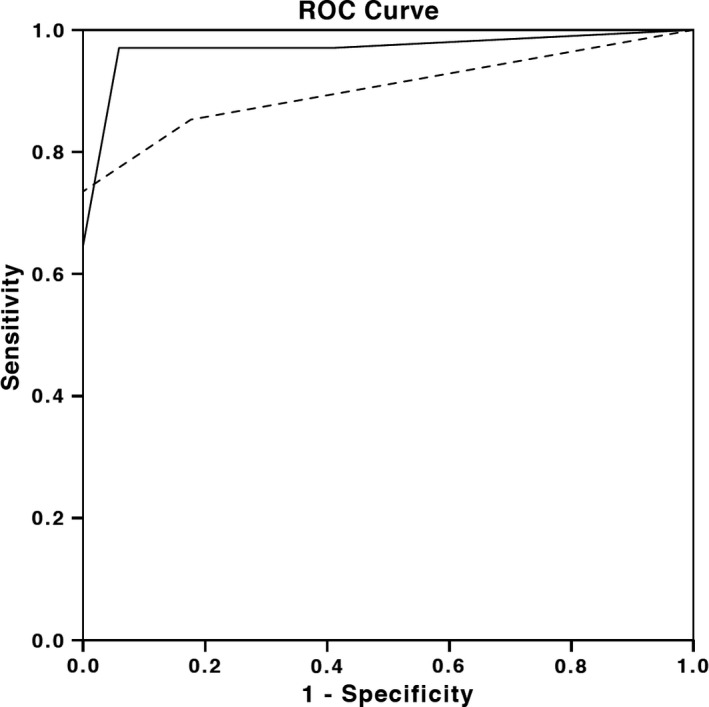
Receiver operating characteristic (ROC) curve for the total number of SNAP asymmetries for the diagnosis of sensory ganglionopathy. Continuous line corresponds to analysis when asymmetry is considered an at least 50% difference in SNAPS/lower SNAP and dotted line corresponds to analysis when asymmetry is considered to be an at least 100% difference in SNAPS/lower SNAP

Among all subjects using a receiver operating characteristic (ROC) curve analysis, as shown in Figure 1, the area under the curve was 0.984 (95% CI, 0.960 to 1.000; SE, 0.012; *p *< .001). In order to detect SG, presence of SNAP asymmetry of >50% in 2 pairs of nerves, not explained by an entrapment neuropathy, shows a sensitivity of 97.1%, a specificity of 94.1%, a positive predictive value of 94.3% and a negative predictive value of 97.0% (Table 2).

**Table 2:** Diagnostic efficiency of the total number of SNAP asymmetries (when asymmetry is considered to be an at least 50% difference in SNAPS/lower SNAP) for the diagnosis of sensory ganglionopathy

Number of asymmetries	Youden index	Sensitivity, %	Specificity, %
1	0.56	97.1	58.8
2	0.91	97.1	94.1
3	0.65	64.7	100
4	0.35	35.3	100
5	0.09	8.8	100

### 3.2 Asymmetry of 100% or more

Among all subjects using a receiver operating characteristic (ROC) curve analysis, as shown in Figure 1, the area under the curve was 0.903 (95% CI, 0.823 to 0.983; SE, 0.041; *p *< .001). In order to detect SG, presence of SNAP asymmetry of >100% in 2 pairs of nerves, not explained by an entrapment neuropathy, shows a sensitivity of 73.5%, a specificity of 100%, a positive predictive value of 100% and a negative predictive value of 79/1% (Table 3).

**Table 3:** Diagnostic efficiency of the total number of SNAP asymmetries (when asymmetry is considered to be an at least 100% difference in SNAPS/lower SNAP) for the diagnosis of sensory ganglionopathy

Number of asymmetries	Youden index	Sensitivity, %	Specificity, %
1	0.68	85.3	82.4
2	0.74	73.5	100
3	0.35	35.3	100
4	0.28	17.6	100
5	0.00	0	100

## 4 DISCUSSION

Early diagnosis of SG is important, especially in paraneoplastic syndromes as an early diagnosis of cancer might improve the clinical outcome. Because of the frequent absence of reference standards, diagnostic criteria of peripheral neuropathies, including SG, have often been established on expert consensus raising the question of whether methodologies independent of subjective appreciations would be more pertinent (Camdessanché et al., 2009). However, this remains a difficult challenge as none of these methods is free of potential bias especially in the selection of the reference and control populations.

In order to limit this bias, in this study we included patients with SG due to conditions known to cause SG (such as gluten sensitivity, Sjögren's syndrome, paraneoplastic disease etc.) who presented with the typical clinical picture of SG (asymmetric patchy sensory deficit and/or rapidly progressing sensory ataxia in the absence of cerebellar pathology). These patients were thoroughly investigated and were under regular follow‐up. In the majority of the cases repeat nerve conduction studies confirmed the progression of the SG to the point that no SNAPs were recordable when all motor NCS were normal. On the other hand, in the control group we included patients with PN presenting with chronic symptoms following a length‐dependent distribution. These patients were also thoroughly investigated and were under regular follow‐up. In order to simulate a real‐life setting we also included subjects with no PN.

Our study presents a novel screening method for SG. The ROC analysis showed that our method (total number of SNAP asymmetries among 5 pairs of nerves) provides an extremely high area under the curve (0.984).

Advantages of our approach include the following:

1. It is not prone to measurement bias, which is likely to occur when measuring the distances between the point of stimulation and the recording electrode.
2. It does not require normative data – which might differ among laboratories using different techniques and/or equipment
3. It is based purely on the SNAP asymmetries between the two sides (right and left), which mirrors the initial clinical presentation of SG in the majority of cases. The asymmetry in the neurophysiological findings is not included in the published diagnostic criteria (Camdessanché et al., 2009), but the criteria only include the clinical asymmetric sensory deficits.
4. A diagnosis of SG can still be made even in the absence of values below normal limits, as the diagnosis is based on the number asymmetries (often patients with SG at early stages present with asymmetric SNAPs, which are still within normal limits).

In our study we matched the control subjects for age and sex in order to limit the possible confounding effect of these variables. Of course, our results should be interpreted with some caution given the fact that our population comprised patients in a unit with an interest in neuropathies, and results may not be generalizable to other settings. The next step to confirm our findings should be a multicenter replication validation study, which could also include other forms of neuropathies in the control groups, both common (i.e. diabetic) and rare (i.e. amyloid).

Choosing the optimal cutoff point is difficult (Budczies et al., 2012). Also deciding on the point beyond which a difference between the left and the right SNAP is considered significant is arbitrary. However, when evaluating a screening tool, a balance between sensitivity and specificity is necessary, as the tool should not only correctly diagnose the condition when present (sensitivity) but also correctly exclude the presence of the condition when not present (specificity). Using the Youden index we found that the best combination of sensitivity and specificity is achieved when asymmetry is considered an at least 50% difference of SNAPs/lower SNAP at the cut‐off point of 2 asymmetries. There, our technique shows a sensitivity of 97.1% and a specificity of 94.1%. However, at a cut‐off point of 3 asymmetries (not explained by entrapment neuropathies) the specificity achieves 100%, meaning that those patients will be definitely suffering from SG. Therefore, our suggestion is to obtain measurements of all pairs of sensory nerves mentioned above, even if there are asymmetries in the first 2 pairs tested.

Similarly high sensitivity (of 100%) is achieved when asymmetry is considered an at least 100% difference of SNAPs/lower SNAP at the cut‐off point of 2 asymmetries. Therefore, the higher the asymmetry is in at least 2 pairs of sensory nerves, the more likely the patient to be suffering from sensory ganglionopathy.

Apart from the diagnostic clinical criteria for confirming SG (Camdessanché et al., 2009) the ulnar sensory‐motor amplitude ratio (Garcia et al., 2013) and the proximally evoked soleus H‐reflex (Zhu et al., 2013) have also been described as neurophysiological methods for screening for SG. A direct comparison of the sensitivity and specificity of our method with those two would be of interest as a future research project.

In our study we investigated the sensitivity and specificity of the number of pairs of SNAPs showing asymmetry for diagnosing SG. All of our cases met the published criteria for SG. A prospective follow‐up study of patients with mild sensory symptoms, without a confirmed diagnosis of SG, with repeat electrophysiological assessment would shed light into the diagnostic ability of our approach in such cases as well as will highlight the natural history of SG and the number and the frequency of electrophysiological assessments needed to confirm a diagnosis of SG.

Clearly, a diagnosis of SG should not be based only on neurophysiological criteria, however it is crucial for the neurophysiologists to be able to provide as much information as possible to the referring physicians in order to make an accurate diagnosis. A rapid screening tool, as the one we are introducing, may be used in identifying subjects very likely to suffer from SG and – in such cases – proceed in further investigations and regular neurological follow‐up to confirm the underlying cause.

## CONFLICT OF INTEREST

None declared.
